# Erratum: Accounting for stimulations that do not elicit motor-evoked potentials when mapping cortical representations of multiple muscles

**DOI:** 10.3389/fnhum.2022.1089615

**Published:** 2022-11-17

**Authors:** Frontiers Production Office

**Affiliations:** Frontiers Media SA, Lausanne, Switzerland

**Keywords:** TMS, motor evoked potential (MEP), muscle mapping, cortical representation, primary motor cortex (M1)

Due to a production error, the incorrect supplementary material was linked to the article.

The publisher apologizes for this mistake. The original version of this article has been updated.

