# Supplementary material for: Accounting for Stimulations That Do Not Elicit Motor-Evoked Potentials When Mapping Cortical Representations of Multiple Muscles
Source: Front Hum Neurosci. 2022 Jun 24;16:920538. doi: 10.3389/fnhum.2022.920538 (PMC9263445; doi:10.3389/fnhum.2022.920538)
Supplement: Supplementary file 1 [file Data_Sheet_1.PDF]

Stimulations that elicited MEPs

Table S1. Overview of elicited MEPs in all the 20 subjects × 8 muscles × 3 intensities × 2 sessions=960 mappings; 'A' stands for active, and' N' stands for non-active.

	Intensity of 105% RMT of FDI															
	Session1								Session2							
	FDI	ADM	APB	FPB	EDC	FDS	ECR	FCR	FDI	ADM	APB	FPB	EDC	FDS	ECR	FCR
**Subject01**	'A'	'A'	'A'	'A'	'A'	'A'	'A'	'A'	'A'	'A'	'A'	'A'	'A'	'A'	'A'	'A'
**Subject02**	'A'	'A'	'A'	'A'	'A'	'A'	'A'	'A'	'A'	'A'	'A'	'A'	'A'	'A'	'A'	'A'
**Subject03**	'A'	'A'	'A'	'A'	'A'	'A'	'A'	'A'	'A'	'A'	'A'	'A'	'A'	'A'	'A'	'A'
**Subject04**	'A'	'A'	'A'	'A'	'A'	'A'	'A'	'A'	'A'	'A'	'A'	'A'	'A'	'A'	'A'	'A'
**Subject05**	'A'	'A'	'A'	'A'	'A'	'A'	'A'	'A'	'A'	'A'	'A'	'A'	'A'	'A'	'A'	'A'
**Subject06**	'A'	'A'	'A'	'A'	'A'	'A'	'A'	'A'	'A'	'A'	'A'	'A'	'A'	'A'	'A'	'A'
**Subject07**	'A'	'A'	'A'	'A'	'A'	'A'	'A'	'A'	'A'	'A'	'A'	'A'	'A'	'A'	'A'	'A'
**Subject08**	'A'	'A'	'A'	'A'	'A'	'A'	'A'	'A'	'A'	'A'	'A'	'A'	'A'	'A'	'A'	'A'
**Subject09**	'A'	'A'	'A'	'A'	'A'	'A'	'A'	'A'	'A'	'A'	'A'	'A'	'A'	'A'	'A'	'A'
**Subject10**	'A'	'A'	'A'	'A'	'A'	'A'	'A'	'A'	'A'	'A'	'A'	'A'	'A'	'A'	'A'	'A'
**Subject11**	'A'	'A'	'A'	'A'	'A'	'A'	'A'	'A'	'A'	'A'	'A'	'A'	'A'	'A'	'A'	'A'
**Subject12**	'A'	'A'	'A'	'A'	'A'	'A'	'A'	'A'	'A'	'A'	'A'	'A'	'A'	'A'	'A'	'A'
**Subject13**	'A'	'A'	'A'	'A'	'A'	'A'	'A'	'A'	'A'	'A'	'A'	'A'	'A'	'A'	'A'	'A'
**Subject14**	'A'	'A'	'A'	'A'	'A'	'A'	'A'	'A'	'A'	'N'	'A'	'A'	'A'	'A'	'A'	'A'
**Subject15**	'A'	'A'	'A'	'A'	'A'	'A'	'A'	'A'	'A'	'A'	'A'	'A'	'A'	'A'	'A'	'A'
**Subject16**	'A'	'A'	'A'	'A'	'A'	'A'	'A'	'A'	'A'	'A'	'A'	'A'	'A'	'A'	'A'	'A'
**Subject17**	'A'	'A'	'A'	'A'	'A'	'A'	'A'	'A'	'A'	'A'	'A'	'A'	'A'	'A'	'A'	'A'
**Subject18**	'A'	'N'	'A'	'A'	'A'	'N'	'A'	'A'	'A'	'A'	'A'	'A'	'A'	'A'	'A'	'A'
**Subject19**	'A'	'A'	'A'	'A'	'A'	'A'	'A'	'A'	'A'	'A'	'A'	'A'	'A'	'A'	'A'	'A'
**Subject20**	'N'	'N'	'N'	'N'	'N'	'N'	'N'	'N'	'A'	'A'	'A'	'A'	'A'	'A'	'A'	'A'

	Intensity of 105% RMT of EDC															
	Session1								Session2							
	FDI	ADM	APB	FPB	EDC	FDS	ECR	FCR	FDI	ADM	APB	FPB	EDC	FDS	ECR	FCR
**Subject01**	'A'	'A'	'A'	'A'	'A'	'A'	'A'	'A'	'A'	'A'	'A'	'A'	'A'	'A'	'A'	'A'
**Subject02**	'A'	'A'	'A'	'A'	'A'	'A'	'A'	'A'	'A'	'A'	'A'	'A'	'A'	'A'	'A'	'A'
**Subject03**	'A'	'A'	'A'	'A'	'A'	'A'	'A'	'A'	'A'	'A'	'A'	'A'	'A'	'A'	'A'	'A'
**Subject04**	'A'	'A'	'A'	'A'	'A'	'A'	'A'	'A'	'A'	'A'	'A'	'A'	'A'	'A'	'A'	'A'
**Subject05**	'A'	'A'	'A'	'A'	'A'	'A'	'A'	'A'	'A'	'A'	'A'	'A'	'A'	'A'	'A'	'A'
**Subject06**	'A'	'A'	'A'	'A'	'A'	'A'	'A'	'A'	'A'	'A'	'A'	'A'	'A'	'A'	'A'	'A'
**Subject07**	'A'	'A'	'A'	'A'	'A'	'A'	'A'	'A'	'A'	'A'	'A'	'A'	'A'	'A'	'A'	'A'
**Subject08**	'A'	'A'	'A'	'A'	'A'	'A'	'A'	'A'	'A'	'A'	'A'	'A'	'A'	'A'	'A'	'A'
**Subject09**	'A'	'A'	'A'	'A'	'A'	'A'	'A'	'A'	'A'	'A'	'A'	'A'	'A'	'A'	'A'	'A'
**Subject10**	'A'	'A'	'A'	'A'	'A'	'A'	'A'	'A'	'A'	'A'	'A'	'A'	'A'	'A'	'A'	'A'
**Subject11**	'A'	'A'	'A'	'A'	'A'	'A'	'A'	'N'	'A'	'A'	'A'	'A'	'A'	'A'	'A'	'A'
**Subject12**	'A'	'N'	'A'	'A'	'A'	'A'	'A'	'A'	'A'	'N'	'A'	'A'	'A'	'A'	'A'	'A'
**Subject13**	'A'	'A'	'A'	'A'	'A'	'A'	'A'	'A'	'A'	'A'	'A'	'A'	'A'	'A'	'A'	'A'
**Subject14**	'A'	'N'	'A'	'A'	'A'	'A'	'A'	'A'	'A'	'N'	'A'	'A'	'A'	'A'	'A'	'A'
**Subject15**	'A'	'A'	'A'	'A'	'A'	'A'	'A'	'A'	'A'	'A'	'A'	'A'	'A'	'A'	'A'	'A'
**Subject16**	'A'	'A'	'A'	'A'	'A'	'A'	'A'	'A'	'A'	'A'	'A'	'A'	'A'	'A'	'A'	'A'
**Subject17**	'A'	'A'	'A'	'A'	'A'	'A'	'A'	'A'	'A'	'A'	'A'	'A'	'A'	'A'	'A'	'A'
**Subject18**	'A'	'A'	'A'	'A'	'A'	'A'	'A'	'A'	'A'	'A'	'A'	'A'	'A'	'A'	'A'	'A'
**Subject19**	'A'	'A'	'A'	'A'	'A'	'A'	'A'	'A'	'A'	'A'	'A'	'A'	'A'	'A'	'A'	'A'
**Subject20**	'A'	'A'	'A'	'A'	'A'	'A'	'A'	'A'	'A'	'A'	'A'	'A'	'A'	'A'	'A'	'A'

	Intensity of 105% RMT of FCR															
	Session1								Session2							
	FDI	ADM	APB	FPB	EDC	FDS	ECR	FCR	FDI	ADM	APB	FPB	EDC	FDS	ECR	FCR
**Subject01**	'A'	'A'	'A'	'A'	'A'	'A'	'A'	'A'	'A'	'A'	'A'	'A'	'A'	'A'	'A'	'A'
**Subject02**	'A'	'A'	'A'	'A'	'A'	'A'	'A'	'A'	'A'	'A'	'A'	'A'	'A'	'A'	'A'	'A'
**Subject03**	'A'	'A'	'A'	'A'	'A'	'A'	'A'	'A'	'A'	'A'	'A'	'A'	'A'	'A'	'A'	'A'
**Subject04**	'A'	'A'	'A'	'A'	'A'	'A'	'A'	'A'	'A'	'A'	'A'	'A'	'A'	'A'	'A'	'A'
**Subject05**	'A'	'A'	'A'	'A'	'A'	'A'	'A'	'A'	'A'	'A'	'A'	'A'	'A'	'A'	'A'	'A'
**Subject06**	'A'	'A'	'A'	'A'	'A'	'A'	'A'	'A'	'A'	'A'	'A'	'A'	'A'	'A'	'A'	'A'
**Subject07**	'A'	'A'	'A'	'A'	'A'	'A'	'A'	'A'	'A'	'A'	'A'	'A'	'A'	'A'	'A'	'A'
**Subject08**	'A'	'A'	'A'	'A'	'A'	'A'	'A'	'A'	'A'	'A'	'A'	'A'	'A'	'A'	'A'	'A'
**Subject09**	'A'	'A'	'A'	'A'	'A'	'A'	'A'	'A'	'A'	'A'	'A'	'A'	'A'	'A'	'A'	'A'
**Subject10**	'A'	'A'	'A'	'A'	'A'	'A'	'A'	'A'	'A'	'A'	'A'	'A'	'A'	'A'	'A'	'A'
**Subject11**	'A'	'A'	'A'	'A'	'A'	'A'	'A'	'A'	'A'	'A'	'A'	'A'	'A'	'A'	'A'	'A'
**Subject12**	'A'	'N'	'A'	'A'	'A'	'A'	'A'	'A'	'A'	'A'	'A'	'A'	'A'	'A'	'A'	'A'
**Subject13**	'A'	'A'	'A'	'A'	'A'	'A'	'A'	'A'	'A'	'A'	'A'	'A'	'A'	'A'	'A'	'A'
**Subject14**	'A'	'A'	'A'	'A'	'A'	'A'	'A'	'A'	'A'	'A'	'A'	'A'	'A'	'A'	'A'	'A'
**Subject15**	'A'	'A'	'A'	'A'	'A'	'A'	'A'	'A'	'A'	'A'	'A'	'A'	'A'	'A'	'A'	'A'
**Subject16**	'A'	'A'	'A'	'A'	'A'	'A'	'A'	'A'	'A'	'A'	'A'	'A'	'A'	'A'	'A'	'A'
**Subject17**	'A'	'A'	'A'	'A'	'A'	'A'	'A'	'A'	'A'	'A'	'A'	'A'	'A'	'A'	'A'	'A'
**Subject18**	'A'	'A'	'A'	'A'	'A'	'A'	'A'	'A'	'A'	'A'	'A'	'A'	'A'	'A'	'A'	'A'
**Subject19**	'A'	'A'	'A'	'A'	'A'	'A'	'A'	'A'	'A'	'A'	'A'	'A'	'A'	'A'	'A'	'A'
**Subject20**	'A'	'A'	'A'	'A'	'A'	'A'	'A'	'A'	'A'	'A'	'A'	'A'	'A'	'A'	'A'	'A'

Number of excitable (active) points

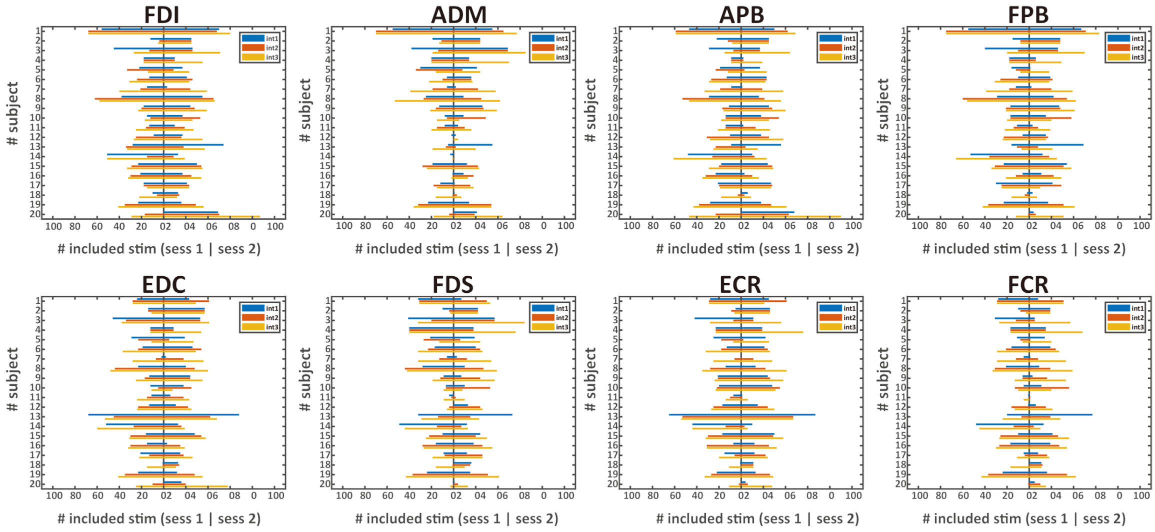

Figure S1. The figure depicts the number of active points for all the muscles in all the subjects. The x-axis values show the number of stimulations in session 1 (centre to the left) and session 2 (centre to the right). Y-axis values represent the number of subjects. The blue, red, and yellow colour legends show the intensity 1,2 and 3 values, respectively.

Standard deviation at baseline

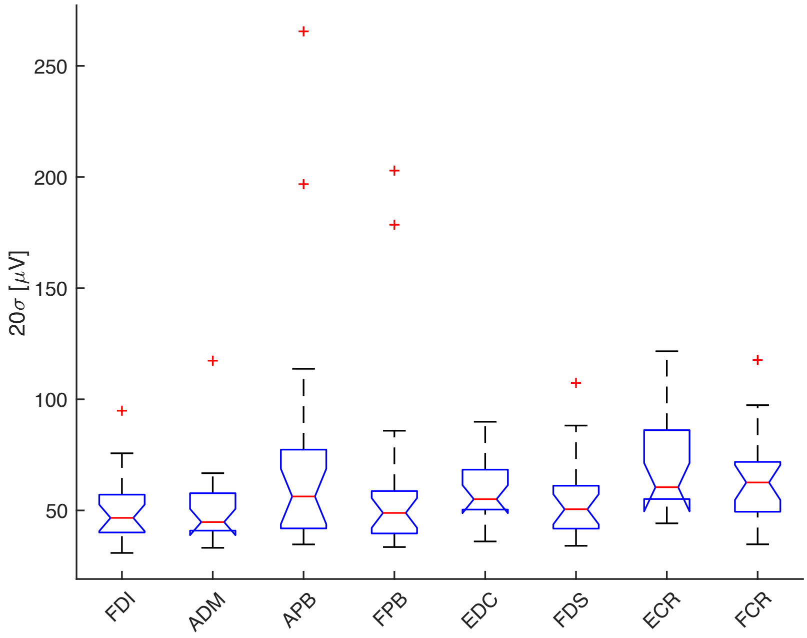

Figure S2. Standard deviations σ per muscle (mean over stimulations and session per subject) that served as threshold to define proper MEPs – see body text. While the median values fall in the range of values reported in the literature (medians of $20 \cdot \sigma$ = [47, 45, 56, 49, 55, 51, 60, and 63] µV for FDI, ADM, APB, FPB, EDC, FDS, ECR, and FCR, respectively), some participants clearly appeared as outliers rendering our choice for subject-specific thresholding via the baseline's standard deviation appropriate.

Supplementary statistics

Table S2a. ICC values of area sizes A and centroids $C = \left(C_x, C_y, C_z\right)^T$ estimated for intensity of 105% RMT of FCR using the cortical meshes with maximum resolution when ignoring non-MEP points (M1) or removing them (M2).*

	FDI		ADM		APB		FPB		EDC		FDS		ECR		FCR	
	M1	M2	M1	M2	M1	M2	M1	M2	M1	M2	M1	M2	M1	M2	M1	M2
Resolution: 15,000 vertices																
A	0.28	0.43	0.65	0.75	0.67	0.45	0.86	0.60	0.76	0.73	0.33	0.30	0.72	0.61	0.24	0.23
C_x	0.90	0.89	0.89	0.89	0.89	0.87	0.91	0.90	0.92	0.91	0.90	0.89	0.93	0.93	0.93	0.92
C_y	0.62	0.60	0.53	0.53	0.61	0.59	0.58	0.56	0.61	0.59	0.55	0.52	0.61	0.57	0.59	0.49
C_z	0.82	0.80	0.72	0.71	0.75	0.72	0.79	0.78	0.81	0.80	0.70	0.70	0.81	0.79	0.82	0.77
Resolution: 100,000 vertices																
A	0.36	0.39	0.72	0.74	0.56	0.55	0.77	0.75	0.84	0.84	0.49	0.47	0.76	0.76	0.31	0.28
C_x	0.90	0.90	0.89	0.89	0.89	0.89	0.88	0.88	0.91	0.91	0.88	0.88	0.92	0.92	0.91	0.91
C_y	0.57	0.57	0.54	0.54	0.67	0.67	0.62	0.62	0.61	0.60	0.51	0.49	0.52	0.51	0.55	0.54
C_z	0.82	0.83	0.69	0.68	0.77	0.79	0.81	0.81	0.78	0.78	0.67	0.67	0.79	0.79	0.85	0.85

* Excellent: .8 ≤ ICC (dark green, bold); good: .65 ≤ ICC < .8 (light green); moderate: .5 ≤ ICC < .65 (yellow); poor: ICC<.5 (light red).

Table S2b. ICC values of area sizes A and centroids $C = \left(C_x, C_y, C_z\right)^T$ estimated for intensity of 105% RMT of FDI using the cortical meshes with maximum resolution when ignoring non-MEP points (M1) or removing them (M2).*

	FDI		ADM		APB		FPB		EDC		FDS		ECR		FCR	
	M1	M2	M1	M2	M1	M2	M1	M2	M1	M2	M1	M2	M1	M2	M1	M2
Resolution: 15,000 vertices																
A	0.64	0.63	0.26	0.32	0.71	0.74	0.40	0.39	0.47	0.16	0.51	0.54	0.60	0.45	0.45	0.44
C_x	0.92	0.91	0.90	0.90	0.87	0.87	0.87	0.86	0.95	0.96	0.94	0.93	0.94	0.94	0.93	0.93
C_y	0.67	0.66	0.51	0.48	0.68	0.69	0.70	0.70	0.64	0.63	0.64	0.64	0.64	0.63	0.68	0.67
C_z	0.82	0.81	0.80	0.79	0.74	0.73	0.71	0.71	0.79	0.76	0.82	0.82	0.81	0.79	0.74	0.73
Resolution: 100,000 vertices																
A	0.57	0.56	0.09	0.08	0.71	0.70	0.44	0.44	0.40	0.32	0.48	0.51	0.64	0.55	0.38	0.37
C_x	0.89	0.89	0.86	0.86	0.85	0.85	0.84	0.85	0.96	0.95	0.90	0.90	0.95	0.95	0.89	0.88
C_y	0.75	0.75	0.61	0.55	0.71	0.71	0.70	0.70	0.69	0.67	0.70	0.70	0.75	0.70	0.65	0.61
C_z	0.83	0.83	0.84	0.84	0.77	0.77	0.73	0.73	0.86	0.86	0.80	0.79	0.86	0.78	0.77	0.80
Maximum resolution																
A	0.46	0.46	0.10	0.06	0.61	0.61	0.48	0.48	0.51	0.49	0.30	0.31	0.60	0.57	0.34	0.29
C_x	0.88	0.89	0.86	0.86	0.85	0.85	0.85	0.85	0.94	0.94	0.89	0.89	0.93	0.93	0.87	0.87
C_y	0.75	0.75	0.59	0.75	0.69	0.69	0.67	0.67	0.67	0.66	0.69	0.69	0.72	0.67	0.62	0.57
C_z	0.80	0.80	0.81	0.82	0.74	0.74	0.70	0.71	0.83	0.83	0.77	0.77	0.82	0.76	0.70	0.72

* Excellent: .8 ≤ ICC (dark green, bold); good: .65 ≤ ICC < .8 (light green); moderate: .5 ≤ ICC < .65 (yellow); poor: ICC<.5 (light red).

Table S2c. ICC values of area sizes A and centroids $C = \left(C_x, C_y, C_z\right)^T$ estimated for intensity of 105% RMT of EDC using the cortical meshes with maximum resolution when ignoring non-MEP points (M1) or removing them (M2).*

	FDI		ADM		APB		FPB		EDC		FDS		ECR		FCR	
	M1	M2	M1	M2	M1	M2	M1	M2	M1	M2	M1	M2	M1	M2	M1	M2
Resolution: 15,000 vertices																
A	0.74	0.66	0.68	0.59	0.48	0.55	0.76	0.78	0.55	0.53	0.55	0.52	0.84	0.82	0.50	0.52
C_x	0.92	0.93	0.89	0.89	0.93	0.93	0.92	0.93	0.88	0.88	0.89	0.90	0.89	0.90	0.87	0.87
C_y	0.52	0.56	0.46	0.46	0.63	0.64	0.73	0.75	0.64	0.64	0.51	0.52	0.62	0.64	0.61	0.61
C_z	0.75	0.75	0.64	0.66	0.76	0.77	0.71	0.88	0.78	0.79	0.75	0.75	0.77	0.79	0.73	0.73
Resolution: 100,000 vertices																
A	0.77	0.75	0.48	0.32	0.50	0.51	0.79	0.81	0.23	0.19	0.37	0.37	0.74	0.73	0.23	0.22
C_x	0.94	0.94	0.87	0.86	0.96	0.95	0.95	0.95	0.90	0.91	0.90	0.90	0.92	0.92	0.88	0.88
C_y	0.58	0.59	0.54	0.60	0.51	0.50	0.77	0.77	0.37	0.63	0.52	0.50	0.68	0.68	0.66	0.67
C_z	0.78	0.77	0.60	0.62	0.68	0.68	0.67	0.81	0.67	0.74	0.75	0.75	0.70	0.71	0.71	0.72
Maximum resolution																
A	0.75	0.75	0.49	0.38	0.56	0.56	0.75	0.74	0.22	0.19	0.38	0.38	0.78	0.78	0.24	0.23
C_x	0.93	0.93	0.83	0.86	0.95	0.95	0.94	0.95	0.89	0.90	0.90	0.90	0.91	0.91	0.87	0.87
C_y	0.59	0.59	0.52	0.56	0.54	0.55	0.72	0.72	0.41	0.66	0.48	0.48	0.68	0.66	0.68	0.68
C_z	0.77	0.77	0.58	0.57	0.68	0.68	0.67	0.81	0.67	0.74	0.79	0.79	0.69	0.68	0.74	0.74

* Excellent: .8 ≤ ICC (dark green, bold); good: .65 ≤ ICC < .8 (light green); moderate: .5 ≤ ICC < .65 (yellow); poor: ICC<.5 (light red).

Table S3a. Outcome of the two-way ANOVA for the area sizes A (in mm^2·μV·10^5) with factors of *intensity* and *session* when considering the 15,000 mesh resolution and when removing the non-MEP points (M2).*

	$\langle A \rangle$ at 105% RMT			*intensity*		*session*		*intensity × session*		*p*-value pairwise comparison		
	FDI	EDC	FCR	*F*	*p*	*F*	*p*	*F*	*p*	FDI/EDC	FDI/FCR	EDC/FCR
FDI	1.51±0.20	2.32±0.46	3.88±0.99	F(2,36)=4.883	**.03**	F(1,18)=1.128	.30	F(2,36)=0.163	.71	.16	.06	.27
ADM	0.72±0.13	0.91±0.13	1.46±0.35	F(2,30)=4.921	**.03**	F(1,15)=0.854	.37	F(2,30)=0.018	.98	.43	.08	.19
APB	1.55±0.41	1.69±0.36	2.96±0.89	F(2,34)=3.412	.07	F(1,17)=1.819	.20	F(2,34)=1.903	.18	1.00	.16	.29
FPB	1.16±0.24	1.43±0.31	1.82±0.37	F(2,34)=3.866	**.03**	F(1,17)=5.534	**.03**	F(2,34)=0.627	.48	.35	.09	.46
EDC	1.00±0.14	1.01±0.14	1.44±0.26	F(2,34)=4.115	**.04**	F(1,17)=0.265	.61	F(2,34)=0.045	.96	1.00	.14	.13
FDS	0.86±0.16	1.00±0.14	1.39±0.19	F(2,30)=5.594	**.01**	F(1,15)=0.142	.71	F(2,30)=1.163	.32	1.00	**.03**	.06
ECR	1.24±0.20	1.64±0.42	2.02±0.39	F(2,32)=3.520	**.04**	F(1,16)=0.716	.41	F(2,32)=0.241	.79	.48	**.04**	.79
FCR	0.68±0.11	1.00±0.18	1.35±0.19	F(2,32)=6.245	**.01**	F(1,16)=0.662	.43	F(2,32)=1.003	.38	.36	**.01**	.27

* Bold face implies $p < .05$.

Table S3b. Outcome of the two-way ANOVA for the area sizes A (in mm^2·μV·10^5) with factors of *intensity* and *session* when considering the 100,000 mesh resolution and when removing the non-MEP points (M2).*

	$\langle A \rangle$ at 105% RMT			*intensity*		*session*		*intensity × session*		*p*-value pairwise comparison		
	FDI	EDC	FCR	*F*	*p*	*F*	*p*	*F*	*p*	FDI/EDC	FDI/FCR	EDC/FCR
FDI	1.48±0.19	2.30±0.53	4.69±1.52	F(2,36)=3.959	.06	F(1,18)=0.84	.37	F(2,36)=0.372	.56	.25	.12	.31
ADM	0.71±0.15	0.85±0.14	1.74±0.52	F(2,30)=4.261	**.05**	F(1,15)=1.054	.32	F(2,30)=0.203	.82	1.00	.09	.25
APB	1.44±0.35	1.75±0.41	3.63±1.26	F(2,34)=3.345	.08	F(1,17)=1.397	.25	F(2,34)=1.444	.25	1.00	.17	.34
FPB	1.11±0.24	1.41±0.35	2.30±0.67	F(2,34)=3.035	.09	F(1,17)=4.891	**.04**	F(2,34)=1.345	.27	.39	.18	.49
EDC	0.92±0.17	0.87±0.11	1.61±0.34	F(2,34)=5.798	**.02**	F(1,17)=0.04	.85	F(2,34)=0.016	.98	1.00	**.04**	.09
FDS	0.80±0.13	0.93±0.16	1.50±0.23	F(2,30)=6.808	**.00**	F(1,15)=1.021	.33	F(2,30)=1.488	.24	1.00	**.02**	.07
ECR	1.16±0.18	1.50±0.39	2.14±0.46	F(2,30)=3.444	**.05**	F(1,15)=0.249	.63	F(2,30)=0.796	.46	0.88	**.05**	.52
FCR	0.66±0.11	0.92±0.16	1.35±0.19	F(2,30)=6.093	**.01**	F(1,15)=1.666	.22	F(2,30)=1.77	.19	.41	**.01**	.25

* Bold face implies $p < .05$.

Table S4. Outcome of the two-way ANOVA for the C_x (in mm) with factors of *intensity* and *session* when considering the 15,000, 100,000 and maximum mesh resolution and when removing the non-MEP points (M2).*

	$\langle A \rangle$ at 105% RMT			*intensity*		*session*		*intensity × session*		*p*-value pairwise comparison		
	FDI	EDC	FCR	*F*	*p*	*F*	*p*	*F*	*p*	FDI/EDC	FDI/FCR	EDC/FCR
**S4a:** Resolution: 15,000 vertices												
FDI	21.26±2.06	20.71±2.03	20.95±1.95	F(2,36)=0.676	.52	F(1,18)=0.316	.58	F(2,36)=1.273	.29	.52	1.00	1.00
ADM	21.06±2.08	20.81±2.06	20.99±2.13	F(2,30)=0.112	.89	F(1,15)=0.072	.79	F(2,30)=4.687	**.02**	1.00	1.00	1.00
APB	21.02±2.06	20.56±2.00	20.42±2.09	F(2,34)=0.901	.42	F(1,17)=0.008	.93	F(2,34)=1.468	.25	.78	.65	1.00
FPB	20.66±1.93	20.56±2.04	20.44±1.97	F(2,34)=0.105	.90	F(1,17)=0.022	.88	F(2,34)=0.017	.94	1.00	1.00	1.00
EDC	19.74±2.00	19.92±2.01	19.87±1.91	F(2,34)=0.115	.89	F(1,17)=0.688	.42	F(2,34)=1.001	.38	1.00	1.00	1.00
FDS	21.40±2.09	21.07±2.22	21.51±2.06	F(2,30)=0.293	.75	F(1,15)=0.127	.73	F(2,30)=0.011	.99	1.00	1.00	1.00
ECR	19.86±2.06	19.43±2.12	19.40±2.14	F(2,32)=0.631	.54	F(1,16)=0.229	.64	F(2,32)=1.054	.36	1.00	.91	1.00
FCR	20.32±2.15	20.46±1.99	20.82±2.12	F(2,32)=0.365	.70	F(1,16)=0.093	.76	F(2,32)=0.871	.43	1.00	1.00	1.00
**S4b:** Resolution: 100,000 vertices												
FDI	21.62±2.09	20.91±1.95	21.03±2.03	F(2,36)=1.618	.21	F(1,18)=0.044	.84	F(2,36)=1.734	.19	.16	.71	1.00
ADM	21.16±2.16	20.95±2.06	21.14±2.04	F(2,30)=0.152	.86	F(1,15)=0.041	.84	F(2,30)=1.968	.16	1.00	1.00	1.00
APB	21.42±2.13	20.96±2.07	20.23±2.09	F(2,34)=3.04	.06	F(1,17)=0.019	.89	F(2,34)=2.077	.14	.93	.13	.43
FPB	20.88±2.03	20.53±2.07	20.31±1.95	F(2,34)=0.733	.49	F(1,17)=0.014	.91	F(2,34)=0.221	.80	1.00	.83	1.00
EDC	20.14±2.08	19.94±2.06	19.84±1.99	F(2,34)=0.658	.52	F(1,17)=0.41	.53	F(2,34)=0.054	.95	1.00	.93	1.00
FDS	21.72±2.10	21.24±2.25	21.30±2.04	F(2,30)=0.396	.68	F(1,15)=0.014	.91	F(2,30)=0.043	.96	1.00	1.00	1.00
ECR	20.46±2.27	20.23±2.25	20.22±2.2	F(2,30)=0.227	.80	F(1,15)=0.08	.78	F(2,30)=0.629	.54	1.00	1.00	1.00
FCR	20.15±2.29	20.56±2.12	20.57±2.28	F(2,30)=0.359	.70	F(1,15)=0.037	.85	F(2,30)=0.161	.85	1.00	1.00	1.00
**S4c:** Maximum resolution												
FDI	21.08±2.07	20.59±1.94	20.95±2.03	F(2,36)=0.677	.52	F(1,18)=0.012	.91	F(2,36)=0.825	.45	.46	1.00	1.00
ADM	20.51±2.60	20.07±2.49	20.18±2.45	F(2,24)=0.477	.63	F(1,12)=0.558	.47	F(2,24)=1.128	.34	1.00	1.00	1.00
APB	21.15±2.04	20.58±2.02	20.22±2.08	F(2,34)=1.668	.20	F(1,17)=0.026	.87	F(2,34)=1.235	.30	.63	.38	1.00
FPB	20.59±2.07	20.33±2.06	20.28±1.97	F(2,34)=0.238	.79	F(1,17)=0.002	.97	F(2,34)=0.153	.86	1.00	1.00	1.00
EDC	19.94±2.07	19.50±2.03	19.69±1.99	F(2,34)=0.674	.52	F(1,17)=0.733	.40	F(2,34)=0.395	.68	.82	1.00	1.00
FDS	21.78±2.13	20.94±2.20	21.41±2.08	F(2,30)=1.031	.37	F(1,15)=0.022	.88	F(2,30)=0.204	.82	.52	1.00	1.00
ECR	20.44±2.27	19.81±2.20	20.12±2.23	F(2,30)=0.884	.42	F(1,15)=0.065	.80	F(2,30)=0.27	.77	.55	1.00	1.00
FCR	20.17±2.24	20.22±2.11	20.60±2.24	F(2,30)=0.321	.73	F(1,15)=0.032	.86	F(2,30)=0.28	.76	1.00	1.00	1.00

* Bold face implies $p < .05$.

Table S5.Outcome of the two-way ANOVA for the C_y (in mm) with factors of *intensity* and *session* when considering the 15,000, 100,000 and maximum mesh resolution and when removing the non-MEP points (M2).*

	$\langle A \rangle$ at 105% RMT			*intensity*		*session*		*intensity × session*		*p*-value pairwise comparison		
	FDI	EDC	FCR	*F*	*p*	*F*	*p*	*F*	*p*	FDI/EDC	FDI/FCR	EDC/FCR
**S5a:** Resolution: 15,000 vertices												
FDI	30.94±1.27	29.80±1.27	30.23±1.05	F(2,36)=1.026	.37	F(1,18)=0.176	.68	F(2,36)=3.644	**.04**	.60	1.00	1.00
ADM	30.17±1.65	29.20±1.56	29.84±1.51	F(2,30)=0.479	.62	F(1,15)=0.65	.43	F(2,30)=1.901	.17	1.00	1.00	1.00
APB	31.58±1.62	30.00±1.41	30.23±1.35	F(2,34)=2.001	.15	F(1,17)=0.006	.94	F(2,34)=3.793	**.05**	.38	.30	1.00
FPB	30.56±1.67	30.20±1.52	30.34±1.27	F(2,34)=0.057	.90	F(1,17)=0.252	.62	F(2,34)=1.7	.21	1.00	1.00	1.00
EDC	29.44±1.43	29.79±1.45	29.51±1.27	F(2,34)=0.100	.91	F(1,17)=0.258	.62	F(2,34)=1.26	.30	1.00	1.00	1.00
FDS	29.77±1.41	28.73±1.51	28.97±1.20	F(2,30)=0.658	.53	F(1,15)=0.041	.84	F(2,30)=0.57	.57	1.00	1.00	1.00
ECR	29.55±1.61	29.19±1.64	29.11±1.36	F(2,32)=0.144	.87	F(1,16)=0.255	.62	F(2,32)=2.316	.12	1.00	1.00	1.00
FCR	29.68±1.48	29.38±1.37	28.63±1.25	F(2,32)=0.587	.56	F(1,16)=0.058	.81	F(2,32)=0.764	.47	1.00	.68	1.00
**S5b:** Resolution: 100,000 vertices												
FDI	31.32±1.41	30.07±1.36	30.87±1.09	F(2,36)=1.226	.31	F(1,18)=0.04	.84	F(2,36)=3.657	**.04**	.45	1.00	.99
ADM	30.27±1.86	29.55±1.64	30.35±1.53	F(2,30)=0.426	.66	F(1,15)=0.196	.66	F(2,30)=1.713	.20	1.00	1.00	1.00
APB	31.78±1.66	30.94±1.46	30.56±1.41	F(2,34)=0.905	.41	F(1,17)=0.068	.80	F(2,34)=2.137	.13	1.00	.58	1.00
FPB	30.64±1.73	30.33±1.49	30.46±1.35	F(2,34)=0.046	.90	F(1,17)=0.069	.80	F(2,34)=1.071	.33	1.00	1.00	1.00
EDC	29.73±1.42	30.30±1.48	29.58±1.28	F(2,34)=0.487	.62	F(1,17)=0.652	.43	F(2,34)=2.032	.15	1.00	1.00	.97
FDS	30.34±1.44	29.49±1.55	29.64±1.15	F(2,30)=0.538	.59	F(1,15)=0.104	.75	F(2,30)=0.812	.45	.96	1.00	1.00
ECR	28.83±1.61	29.12±1.68	29.06±1.31	F(2,30)=0.058	.94	F(1,15)=0.109	.75	F(2,30)=2.469	.10	1.00	1.00	1.00
FCR	29.46±1.38	29.84±1.52	29.15±1.27	F(2,30)=0.32	.73	F(1,15)=0.044	.84	F(2,30)=0.241	.79	1.00	1.00	1.00
**S5c:** Maximum resolution												
FDI	31.01±1.41	29.79±1.36	30.28±1.12	F(2,36)=0.994	.38	F(1,18)=0.773	.39	F(2,36)=4.401	**.02**	.42	1.00	1.00
ADM	30.23±2.25	29.23±1.99	29.64±1.76	F(2,24)=0.488	.62	F(1,12)=2.171	.17	F(2,24)=2.021	.15	1.00	1.00	1.00
APB	31.22±1.65	30.58±1.49	30.20±1.40	F(2,34)=0.555	.58	F(1,17)=0.05	.83	F(2,34)=2.018	.15	1.00	.96	1.00
FPB	30.46±1.75	30.44±1.56	30.17±1.34	F(2,34)=0.041	.96	F(1,17)=0.63	.44	F(2,34)=0.596	.56	1.00	1.00	1.00
EDC	29.50±1.47	30.07±1.55	29.29±1.24	F(2,34)=0.478	.62	F(1,17)=0.799	.38	F(2,34)=1.721	.19	1.00	1.00	1.00
FDS	29.74±1.55	28.93±1.62	28.85±1.24	F(2,30)=0.468	.63	F(1,15)=0.575	.46	F(2,30)=1.016	.37	1.00	1.00	1.00
ECR	28.63±1.63	28.68±1.74	28.87±1.33	F(2,30)=0.042	.96	F(1,15)=0.211	.65	F(2,30)=3.445	**.05**	1.00	1.00	1.00
FCR	29.12±1.40	29.64±1.62	29.03±1.27	F(2,30)=0.260	.77	F(1,15)=0.012	.91	F(2,30)=0.795	.46	1.00	1.00	1.00

* Bold face implies $p < .05$.

Table S6.Outcome of the two-way ANOVA for the C_z (in mm) with factors of *intensity* and *session* when considering the 15,000, 100,000 and maximum mesh resolution and when removing the non-MEP points (M2).*

	$\langle A \rangle$ at 105% RMT			*intensity*		*session*		*intensity × session*		*p*-value pairwise comparison		
	FDI	EDC	FCR	*F*	*p*	*F*	*p*	*F*	*p*	FDI/EDC	FDI/FCR	EDC/FCR
**S6a:** Resolution: 15,000 vertices												
FDI	108.18±1.69	108.65±1.72	107.70±1.55	F(2,36)=1.097	.35	F(1,18)=0.065	.80	F(2,36)=2.316	.11	1.00	1.00	.60
ADM	109.11±1.80	109.14±1.93	108.56±1.72	F(2,30)=0.380	.69	F(1,15)=0.144	.71	F(2,30)=0.714	.50	1.00	1.00	1.00
APB	108.19±1.60	108.75±1.58	108.07±1.52	F(2,34)=0.563	.53	F(1,17)=0.035	.85	F(2,34)=1.420	.26	1.00	1.00	1.00
FPB	109.12±1.70	108.14±1.74	108.09±1.71	F(2,34)=1.031	.34	F(1,17)=0.317	.58	F(2,34)=0.849	.44	1.00	.35	1.00
EDC	109.40±1.66	108.55±1.80	108.78±1.65	F(2,34)=0.929	.37	F(1,17)=0.244	.63	F(2,34)=1.099	.35	.89	.18	1.00
FDS	110.07±1.65	109.42±1.89	109.55±1.66	F(2,30)=0.602	.49	F(1,15)=0.004	.95	F(2,30)=0.356	.70	1.00	.41	1.00
ECR	108.92±1.85	108.81±1.70	108.68±1.66	F(2,32)=0.054	.95	F(1,16)=0.012	.91	F(2,32)=0.777	.41	1.00	1.00	1.00
FCR	110.35±1.46	109.51±1.68	109.87±1.57	F(2,32)=0.741	.45	F(1,16)=0.164	.69	F(2,32)=0.732	.49	.96	.85	1.00
**S6b:** Resolution: 100,000 vertices												
FDI	108.70±1.67	109.35±1.79	107.99±1.56	F(2,36)=2.154	.15	F(1,18)=0.074	.79	F(2,36)=3.139	.06	1.00	.37	.23
ADM	109.82±1.85	109.73±1.95	108.75±1.80	F(2,30)=1.312	.28	F(1,15)=0.003	.96	F(2,30)=1.274	.29	1.00	.55	.56
APB	108.90±1.71	109.61±1.66	108.51±1.57	F(2,34)=1.060	.36	F(1,17)=0.079	.78	F(2,34)=1.572	.22	1.00	1.00	.43
FPB	109.83±1.67	109.13±1.79	108.54±1.72	F(2,34)=1.245	.29	F(1,17)=0.000	.98	F(2,34)=0.367	.70	1.00	.22	1.00
EDC	110.42±1.66	109.35±1.86	109.35±1.67	F(2,34)=1.557	.23	F(1,17)=0.324	.58	F(2,34)=0.505	.61	.67	**.04**	1.00
FDS	110.80±1.63	110.44±1.86	109.79±1.61	F(2,30)=1.207	.30	F(1,15)=0.002	.96	F(2,30)=0.542	.59	1.00	.06	1.00
ECR	110.86±1.52	110.37±1.73	110.11±1.60	F(2,30)=0.65	.53	F(1,15)=0.112	.74	F(2,30)=0.577	.57	1.00	.32	1.00
FCR	110.99±1.57	110.19±1.78	110.15±1.67	F(2,30)=0.897	.42	F(1,15)=0.071	.79	F(2,30)=0.281	.76	0.88	.32	1.00
**S6c:** Maximum resolution												
FDI	108.60±1.71	109.26±1.80	108.02±1.53	F(2,36)=1.558	.23	F(1,18)=0.177	.68	F(2,36)=2.879	.07	1.00	.87	.39
ADM	108.92±2.15	108.98±2.31	108.54±2.13	F(2,24)=0.262	.77	F(1,12)=0.366	.56	F(2,24)=1.155	.33	1.00	1.00	1.00
APB	108.92±1.70	109.51±1.66	108.47±1.57	F(2,34)=0.822	.45	F(1,17)=0.048	.83	F(2,34)=0.844	.44	1.00	1.00	.65
FPB	109.78±1.70	109.03±1.80	108.59±1.69	F(2,34)=1.103	.33	F(1,17)=0.022	.88	F(2,34)=0.137	.87	1.00	.26	1.00
EDC	110.22±1.66	109.14±1.84	109.34±1.62	F(2,34)=1.411	.26	F(1,17)=0.281	.60	F(2,34)=0.389	.68	.56	.19	1.00
FDS	110.75±1.63	110.46±1.86	109.96±1.62	F(2,30)=0.663	.47	F(1,15)=0.075	.79	F(2,30)=0.959	.40	1.00	.21	1.00
ECR	110.76±1.53	110.35±1.75	109.72±1.52	F(2,30)=1.242	.30	F(1,15)=0.128	.73	F(2,30)=1.568	.23	1.00	.13	1.00
FCR	110.95±1.54	110.19±1.80	110.15±1.66	F(2,30)=0.810	.42	F(1,15)=0.138	.72	F(2,30)=0.199	.82	1.00	.22	1.00

* Bold face implies $p < .05$.

Table S7. Outcome of the two-way ANOVA for the amplitude (μV) with factors of *intensity* and *session* when considering the 15,000 mesh resolution and when removing the non-MEP points (M2).*

	$\langle A \rangle$ at 105% RMT			*intensity*		*session*		*intensity × session*		p-value pairwise comparison		
	FDI	EDC	FCR	*F*	*p*	*F*	*p*	*F*	*p*	FDI/ EDC	FDI/ FCR	EDC/ FCR
FDI	286.59±38.83	365.52±64.20	561.15±127.18	F(2,36)=5.913	**.02**	F(1,18)=0.513	.48	F(2,36)=0.520	.50	.20	.05	.12
ADM	183.30±34.68	187.29±34.84	260.86±56.30	F(2,30)=5.372	**.03**	F(1,15)=0.064	.80	F(2,30)=0.792	.46	1.00	.07	.12
APB	343.04±84.23	299.11±55.85	407.51±104.16	F(2,34)=1.191	.32	F(1,17)=1.401	.25	F(2,34)=0.645	.53	1.00	1.00	.44
FPB	236.32±38.56	223.33±38.80	278.20±53.89	F(2,34)=2.053	.16	F(1,17)=7.836	**.01**	F(2,34)=2.315	.11	1.00	.70	.25
EDC	170.86±20.53	160.85±12.49	210.73±21.32	F(2,36)=6.413	**.00**	F(1,18)=0.626	.44	F(2,36)=0.352	.71	1.00	.07	**.01**
FDS	168.04±19.51	179.02±23.05	222.47±27.17	F(2,30)=4.572	**.02**	F(1,15)=3.047	.10	F(2,30)=1.604	.22	1.00	.09	.07
ECR	222.46±23.31	247.82±38.31	276.44±37.80	F(2,32)=2.586	.09	F(1,16)=0.320	.58	F(2,32)=0.237	.72	.99	.07	.75
FCR	157.21±13.97	174.40±18.41	203.78±20.27	F(2,32)=4.645	**.02**	F(1,16)=0.068	.80	F(2,32)=0.420	.66	.72	.07	.13

* Bold face implies $p < .05$.

Table S8. The ICC values for measurement are shown in the table for all the muscles. Int 1,2,3 represent the intensities of 105% RMT of FDI, EDC and FCR, respectively. The bold font implies that ICC values are excellent (ICC>0.8).

**FDI**	**Int1**	**Int2**	**Int3**	**ADM**	**Int1**	**Int2**	**Int3**	**APB**	**Int1**	**Int2**	**Int3**	**FPB**	**Int1**	**Int2**	**Int3**
Amp	0.73	**0.83**	0.34	Amp	0.47	0.61	0.75	Amp	0.58	0.59	0.69	Amp	0.53	**0.87**	0.73
Lat	**0.81**	**0.93**	**0.87**	Lat	0.78	0.64	0.66	Lat	0.64	0.42	0.72	Lat	0.27	0.57	0.18
CoG_x	**0.93**	**0.94**	**0.94**	CoG_x	**0.87**	**0.87**	**0.90**	CoG_x	**0.88**	**0.93**	**0.92**	CoG_x	**0.84**	**0.93**	**0.94**
CoG_y	0.74	0.64	0.60	CoG_y	0.57	0.62	0.58	CoG_y	0.75	0.62	0.65	CoG_y	0.66	0.72	0.56
CoG_z	**0.91**	**0.83**	**0.82**	CoG_z	**0.86**	0.78	0.77	CoG_z	**0.84**	0.79	0.79	CoG_z	0.77	**0.87**	**0.80**
**EDC**	**Int1**	**Int2**	**Int3**	**FDS**	**Int1**	**Int2**	**Int3**	**ECR**	**Int1**	**Int2**	**Int3**	**FCR**	**Int1**	**Int2**	**Int3**
Amp	0.76	0.56	0.77	Amp	0.71	0.74	0.42	Amp	**0.84**	**0.94**	0.76	Amp	0.53	0.60	0.30
Lat	0.53	0.40	0.77	Lat	0.46	0.51	0.67	Lat	0.69	**0.82**	**0.84**	Lat	0.45	0.73	0.76
CoG_x	**0.94**	**0.93**	**0.93**	CoG_x	**0.92**	**0.92**	**0.91**	CoG_x	**0.92**	**0.92**	**0.93**	CoG_x	**0.91**	**0.89**	**0.95**
CoG_y	0.75	0.50	0.63	CoG_y	0.72	0.63	0.55	CoG_y	0.75	0.70	0.64	CoG_y	0.68	0.70	0.64
CoG_z	**0.91**	0.75	**0.83**	CoG_z	**0.85**	**0.87**	0.77	CoG_z	**0.91**	0.78	**0.84**	CoG_z	**0.82**	**0.81**	**0.91**
